# Efficacy of rifabutin-based triple therapy as second-line treatment to eradicate helicobacter pylori infection

**DOI:** 10.1186/1471-230X-7-31

**Published:** 2007-07-25

**Authors:** José M Navarro-Jarabo, Nuria Fernández, Francisca L Sousa, Encarnación Cabrera, Manuel Castro, Luz M Ramírez, Robin Rivera, Esther Ubiña, Francisco Vera, Isabel Méndez, Francisco Rivas-Ruiz, José L Moreno, Emilio Perea-Milla

**Affiliations:** 1 Unidad de Aparato Digestivo, Hospital Costa del Sol, Ctra Nacional 340, km 187, 29600 Marbella, Spain; 2 Sección de Aparato Digestivo, Hospital de Motril, Av. Enrique Martín Cuevas, S/N 18600 Motril, Spain; 3 Servicio de Aparato Digestivo, Hospital General de Especialidades de Jaén, Avda. del Ejército Español, 10. 23007 Jaén, Spain; 4 Servicio de Aparato Digestivo, Hospital Universitario Ntra. Sra. de Valme, Ctra. de Cádiz km. 548,9, 41014 Sevilla, Spain; 5 Sección de Aparato Digestivo, Hospital Comarcal Valle de los Pedroches, Juan del Rey Calero s/n, 14400 Pozoblanco, Córdoba, Spain; 6 Unidad de Apoyo a la Investigación, Hospital Costa del Sol, Ctra Nacional 340, km 187, 29600 Marbella, Spain; 7 Unidad de Farmacia, Hospital Costa del Sol, Ctra Nacional 340, km 187, 29600 Marbella, Spain

## Abstract

**Background:**

Rifabutin has been found to be effective in multi-resistant patients after various treatment cycles for Helicobacter pylori (HP) infection, but it has not been analysed as a second-line treatment. Therefore, we seek to compare the effectiveness of a treatment regimen including rifabutin versus conventional quadruple therapy (QT).

**Methods:**

Open clinical trial, randomised and multi-centre, of two treatment protocols: A) Conventional regime -QT- (omeprazole 20 mg bid, bismuth citrate 120 mg qid, tetracycline 500 mg qid and metronidazole 500 mg tid); B) Experimental one -OAR- (omeprazole 20 mg bid, amoxicillin 1 gr bid, and rifabutin 150 mg bid), both taken orally for 7 days, in patients with HP infection for whom first-line treatment had failed. Eradication was determined by Urea Breath Test (UBT). Safety was determined by the adverse events.

**Results:**

99 patients were randomised, QT, n = 54; OAR, n = 45. The two groups were homogeneous. In 8 cases, treatment was suspended (6 in QT and 2 in OAR). The eradication achieved, analysed by ITT, was for QT, 38 cases (70.4%), and for OAR, 20 cases (44.4%); p = 0.009, OR = 1.58. Of the cases analysed PP, QT were 77.1%; OAR, 46.5%; p = 0.002. Adverse effects were described in 64% of the QT patients and in 44% of the OAR patients (p = 0.04).

**Conclusion:**

A 7-day rifabutin-based triple therapy associated to amoxicillin and omeprazole at standard dose was not found to be effective as a second-line rescue therapy. The problem with quadruple therapy lies in the adverse side effects it provokes. We believe the search should continue for alternatives that are more comfortably administered and that are at least as effective, but with fewer adverse side effects.

**Trial Registration:**

Current Controlled Trials ISRCTN81058036

## Background

Helicobacter pylori infection plays an important role in the phatogenesis of chronic gastritis, gastroduodenal ulcer, maltoma and gastric adenocarcinoma. First-line treatment with triple therapy (proton pump inhibitor (PPI)- associated with clarithromycin and amoxicillin or metronidazole) is widely accepted and applied [1,2] and has an effectiveness rate of almost 80%. Nevertheless, a considerable proportion of patients fail to respond to the latter treatment, and for these there is no ideal treatment [3,4]. Quadruptherapy (PPI – associated with bismuth citrate, tetracycline and metronidazole) is the most commonly used second-line treatment, with an eradication rate of 57–95% [4-7]. However, this is not totally satisfactory, due to the complexity of the dosing regimen and the frequency of associated side effects [8]. Thus, alternative options to be applied must be more effective, simpler and better tolerated than the quadruple therapy.

In recent years, it has been shown that some antibiotics can be useful in such circumstances. One of these is rifabutin: derived from rifampicin, it is used as a rescue treatment against mycobacterium tuberculosis and as a prophylactic against the mycobacterium avium intracellulare infection of HIV-positive patients. It has been shown to be effective in eradicating HP [9,10]. In relation to this latter effect, it is very effective in vitro [11-13], achieving lower levels of minimum inhibitory concentration than obtained by clarithromycin and amoxicillin [14]. Moreover, its effectiveness does not depend on the pH of the medium [15].

Many features make rifabutin-based therapy an interesting alternative for clinical application to achieve the eradication of HP. Firstly, it is effective at low doses [16] (300 mg), and so its side effects are minimised [17]. Secondly, the primary resistance of HP to rifabutin in the population at large is non-existent, because this antibiotic is only used for very specific clinical situations [11,18]. Finally, its efficacy is not reduced by the resistance that HP may develop to other antibiotics, especially clarithromycin and metronidazole [16,18,26], and so it can be administered as a rescue treatment without the need for a prior antibiogram.

Rifabutin-based therapy has been applied as a rescue treatment for patients for whom one or more other treatments have failed, i.e. as a rescue regimen for difficult to treat patients [16,10,19,20,26]. No study of it has been made as a second-line treatment, among a homogeneous group of patients for whom a single eradication treatment of triple therapy with IBP, clarithromycin and amoxicillin/metronidazole has failed.

To examine the hypothesis that a regimen including rifabutin may be as effective as one based on quadruple therapy as a rescue treatment, and moreover, that it may achieve higher levels of clinical tolerance, we designed a multicentre, randomised, open clinical trial, with the participation of five hospitals in southern Spain.

## Methods

### Study Design

This study was performed in acordance with the declaration of Helsinki, and was approved by the Clinical Trials Committee of the Autonomous Administration of Andalucía, and by the corresponding committee of Hospital Costa del Sol (Marbella), Hospital de Motril (Granada), Hospital de Especialidades (Jaen), Hospital de Valme (Sevilla) and Hospital Valle de los Pedroches (Pozoblanco).

Patients were allocated to one of the following treatment regimens: A) OAR (Omeprazole 20 mg/12 h, Amoxicillin 1 gr/12 h, Rifabutin 150 mg/12 h); and B) QT (Omeprazole 20 mg/12 h, Bismuth Citrate 120 mg/6 h, Tetracycline 500 mg/6 h, and Metronidazole 500 mg/8 h). In both cases, the medicaments were administered orally, for 7 days. Patients were progressively included in the trial as determined by the application of a random number table, with an entry number that was unknown to both researchers and patients. All patients signed a written informed consent form. The medication provided was not masked, because the main aim of the trial is influenced by adherence to the treatment regimen, which in turn is affected by the ease, or otherwise, with which the treatment is administered. The medication was prepared by the pharmaceutical service of the organising healthcare provider, delivered in a sealed package, with the number of capsules necessary for each group, and distributed to each participating hospital. Each patient included in the study received a clinical examination (or replied to a telephone interview) to identify the side effects caused by the treatment, following a standardised protocol [21]. At 45 days after ending the treatment, a secondary medical review was performed, at which eradication of HP was determined by UBT and, again, side effects were identified. At this moment, surplus medication was returned. Consumption by the patient of less than 90% of the capsules initially provided was evaluated as non-fulfilment of the recommended therapy. The patient was classified as having withdrawn from the study if follow up was not performed.

### Inclusion and exclusion criteria

Patients in whom Helicobacter pylori infection persisted after a triple therapy treatment were included. Persistence of infection was determined by a breath test, by a rapid urease test, by pathological anatomy or by culture. The initial diagnosis of Helicobacter pylori infection was made by invasive methods (rapid ureasa test and/or pathological anatomy and/or Helicobacter culture) or by non-invasive ones, i.e. a urea breath test decided upon after infrared spectrophotometry or mass-spectrometry. All the patients were given an initial endoscopy prior to the first attempt at eradicating the infection. Patients were excluded from the trial if they withheld consent, if they had initially been treated by the "Test and Treat" procedure or if a baseline endoscopy was not obtained. Also excluded were those patients for whom fulfilment of the treatment regimen and attendance at follow-up appointments could not reasonably be expected. Other conditions for exclusion were HIV positive status, active alcoholism, addiction to drugs, age less than 18 years or more than 75 years, the suspicion of tuberculous infection, either because of a positive intradermal reaction to Mantoux and compatible thorax radiography, or if the patient had previously received tuberculostatic treatment, or a known allergy to any of the components of either of the two treatment regimens. Patients who had received quadruple therapy as first-line treatment, or any other treatment including bismuth (e.g., ranitidine bismuth citrate), or antibiotics during the previous month, were excluded from the trial. In addition, patients with severe associated diseases (cardiac insufficiency, respiratory insufficiency, chronic kidney insufficiency, hepatic insufficiency, advanced neoplasic diseases), as well as those who were pregnant or lactating, were excluded.

### Principal variable

Eradication was defined as having occurred when a negative UBT result was recorded, with urea marked with 13-C, at 45 days after finalising the treatment. A minimum of 15 days of suspension of any proton pump inhibitor was required, and a similar period of abstinence from antibiotics before the UBT. The test was carried out by personnel who were unaware of the medication taken by the patient, using the infrared spectrophotometry procedure, which has a diagnostic efficacy similar to that obtained by mass spectrophotometry [22,23] (UbiT -IR 300 – Otsuka Electronics Co., Ltd). The results were considered to be negative when the ratio obtained between the baseline sample and that obtained after the administration of urea marked with 13-C was ≤ 2.5 0/00 and positive if this value was higher than 2.5 0/00. The analysis of efficacy was planned: a) by intention to treat (ITT), on all the patients included in the trial (in the case of loss of follow up, this was considered a positive result in the experimental group and a negative one in the quadruple therapy following the worse case method); b) per protocol (PP), which included only the patients who had completed the whole follow up procedure and had consumed at least 90% of the medication provided.

### Secondary variable

Adverse side effects were recorded on a purpose-made form, at 7 and 45 days after finalising the treatment. Such effects were considered mild if they were well tolerated by the patient, moderate if they were of sufficient intensity as to interfere with the patient's normal life or as to require medical intervention, serious when they impeded the performance of everyday activities, significantly affected the clinical situation and justified medical intervention, and acute when the patient's life was put at risk.

### Sample size

The sample size was calculated according to a forecast eradication rate of 67% with the QT (based on the values obtained in preliminary studies at our Hospital, unpublished data) and on a forecast success rate of 84% for OAR, with a level of significance of 0.05 and a power of 0.80. We foresaw a minimum number of 102 patients per group, this figure including a 10% oversizing element. The trial was designed in the expectation of an intermediate analysis when half of the calculated sample size had been achieved; the trial would be interrupted if a difference equal to or greater than 30% determined by ITT was found, if such a difference were statistically significant.

### Statistical analysis

A descriptive statistical analysis was carried out, with measures of central trend and dispersion for the continuous variables, and frequency distribution for the qualitative variables, comparing the baseline levels of the two groups of patients for the membership variables (age and sex) and clinical variables (type of endoscopic injury, tobacco consumption, defined as smoking 10 or more cigarettes per day). We compared the rate of eradication in each treatment group, using the odds ratio (OR) of incidence, and the chi squared test was used against the hypothesis. A multivariate logistic regression model was built adjusting for basal variables. The level of statistical significance was established as p < 0.05.

## Results

**Figure 1:**
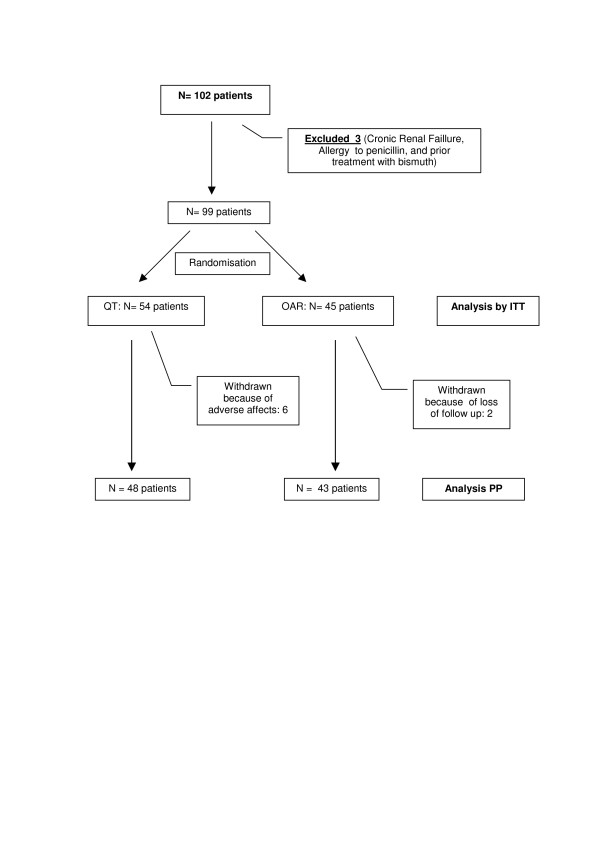
**Inclusion and flow of patients**. ITT: Intention to Treat. PP: Per Protocol.

Patients were included in the study from September 2004 until August 2005, and an intermediate analysis was performed (from which definitive results were obtained) when half of the initially scheduled sample had been examined. Figure 1 shows that of a total of 102 patients, three were excluded.

Of the 99 patients definitively included, 45 were assigned to Group A (OAR) and 54 to Group B (QT). Table 1 shows that the groups were homogeneous with regard to sex, age, tobacco consumption and endoscopic injury. Although there was a higher proportion of ulcerous patients in the QT group, this difference was not statistically significant. The QT group presented a higher consumption of NSAIDs.

**Table 1:** Demographic data of the patients.

	QT (n = 54)	OAR (n = 45)	P value
Age	46.4	48.6	ns
Sex (male/female)	25/29	23/22	ns
Caucasian	96.3%	97.7%	
Endoscopic findings			
Chronic gastritis	25 (46.3%)	28 (62.2%)	
GD Ulcer	29 (53.7%)	17 (37.8%)	ns
Smokers	12 (22.2%)	10 (22.2%)	ns
Alcohol consumers	15 (27.8%)	11 (24.4%)	ns
ASA/NSAID	10 (18.5%)	2 (4.4%)	0.031

GD Ulcer: Gastroduodenal ulcer

Smokers ≥ 10 cigarettes per day

Alcohol consumers ≥ 2 units per day

ns: no significant

Table 2 illustrates the efficacy of the two treatments. By intention to treat (ITT), in the QT group, HP was eradicated in 38 of the 54 patients (70.4%), while the OAR treatment only eradicated it in 20 of the 45 cases (44.4%); the difference was statistically significant (p = 0.009; OR = 1.58). In the per protocol (PP) analysis, similar differences were observed: a cure was achieved in 37 of the 48 patients who completed the treatment (77%), and in 20 of the 43 who finalised the OAR programme (45%); this difference was also statistically significant (p = 0.002). One patient in the QT group achieved HP eradication despite not having completed the course of treatment. The adjustment for the basal variables in the model of logistic regression did not modify these results (data not shown).

**Table 2:** Eradication rates of the study population.

	Intention to Treat			Per Protocol		
UBT	QT	OAR	CI 95%	QT	OAR	CI 95%
Negative	38 (70.4%)	20 (40.4%)	**p = 0.009**	37 (77.1%)	20 (46.5%)	**p = 0.003**
Positive	16 (29.6%)	25 (55.6%)		11 (22.9%)	23 (53.5%)	
N	54	45	**OR 1.58 (1.1–2.29)**	48*	43	**OR 1.65 (1.1–2.36)**

* One patient achieved eradication without having completed the treatment programme

We analysed the efficacy achieved with respect to the endoscopic injury and no differences were found in eradication rates between ulcerous (56.8% of 44 cases) and nonulcerous patients (57.7%).

The treatment was not completed in 8 cases: 6 in the QT group because of unacceptable side effects (UBT control was achieved in all), and 2 in the OAR group, because of loss of follow up.

Table 3 shows the adverse events recorded. In the QT group 35 patients (64%) reported at least one adverse event, versus 44% of the patients in the OAR group (p = 0.042). The most commonly described such events were epigastralgia and/or dyspepsia, taste loss or variation, dizziness, diarrhoea, nausea and/or vomiting, loss of appetite and cephalea. Severe effects were much less frequent. No leucopoenia was found. 14 events were classified as severe; of these, 11 were in the QT group and 3 in the OAR group. No patient in the latter group stopped medication for this reason.

**Table 3:** Side effects in each treatment group.

	*QT*	*OAR*
Total patients	35 (64%) *	20 (44%) *
Dyspepsia		
+	17	8
+++	1	1
Taste-sensation alteration		
+	12	5
+++	2	
Dizziness		
+	13	4
+++	1	
Diarrhoea		
+	10	6
+++	1	
Nausea/Vomiting		
+	10	5
+++	3	1
Loss of appetite		
+	9	6
+++	2	
Cephalea		
+	8	6
+++	1	1
Odynophagia		
+	4 (2 candidiasis)	
+++		
Asthenia/tiredness		
+	2	3
+++		
Total episodes	85	43
Total episodes +++	11	3

* p 0.042

+: : mild/moderate

+++: : severe

## Discussion

A controlled study was carried out to compare a quadruple therapy and an experimental one with rifabutin (associated with omeprazole and amoxicillin), as second-line treatment. The efficacy obtained with the rifabutin regimen was 44% (ITT) and 45% (PP), greatly inferior to that obtained with the quadruple therapy (70% and 76%, respectively).

HP infection is not easy to eradicate when the first-line treatment fails. The most important predictors of failure are antibiotics resistance and regimen compliance. Quadruple therapy has an estimated efficacy of about 75% [24], but is uncomfortable to administer, because of the large number of pills required; moreover, it provokes side effects that have a negative influence on completion of the regimen, in normal clinical practice [8]. For these reasons, many attempts have been made to find alternative treatment regimens that do not present the above problems. Most such options are based on alternative antibiotics, because the repetition of treatment cycles based on the same antibiotics, especially clarithromycin and/or metronidazole, has no possibility whatever of success [25].

In preliminary studies, rifabutin-based therapy had good results in patients with one or more failed attempts at eradication, both for patients who are sensitive to antibiotics and for those who have developed secondary resistance to clarithromycin and metronidazole as a result of previous treatments [16,26]. In such studies, the antibiotic association most often used is rifabutin 300 mg/day and amoxicillin (1 gr/12 h), although tests have also been made with levofloxacin [27].

The present study describes the first clinical trial designed to compare a rifabutin-based treatment, as a second-line rescue treatment, with quadruple therapy, which is the standard recommended. The previous results obtained with rifabutin-based therapy had been very promising, with eradication rates of 82–91% [10,16,19,26,27]. However, our clinical study does not corroborate the above eradication rates and was found to be less effective than the quadruple therapy. The latter, on the other hand, in our series achieved a success rate similar to that found by other authors [24].

The eradication rate obtained with our rifabutin-based regimen is one of the lowest published to date. In the first trial reported, Perri et al. [16] obtained an effectiveness of 80% with a regimen similar to ours, surpassing the 66% obtained with the quadruple therapy. Perhaps the different duration of the treatment implemented (10 days) may partially explain the difference in the results achieved. Other authors, however, have published similar results with rifabutin-based therapy for seven days: Bock [10] reported an efficacy of 71% in a non-comparative prospective study, in combination with amoxicillin and pantoprazole. Longer treatment periods, nevertheless, are not reported to improve success rates: Gisbert [19] obtained an eradication rate of 79% in a series limited to 14 cases, as a third-line rescue treatment, in which the regimen was administered for two weeks. Torachio [26] published a study of a 10 day regimen with amoxicillin and pantoprazole, applied only to patients with resistance to clarithromycin and metronidazole (whether primary or induced by previous eradication treatment). An eradication rate of 87% was achieved among the patients who had not been treated previously and one of 78.5% among those with a history of failed prior eradication. Recently Borody [28] has reported an eradication rate of 96.6% with a lower dose of rifabutin (150 mg per day) and an increase in the dose of amoxicillyn (1.5 gr. tid) and PPI's (pantoprazole 80 mg tid) for 12 days.

Associated with antibiotics other than amoxicillin, Wong [27] published a comparative study with quadruple therapy applied to patients with one or more previous attempts at eradication, associating rifabutin (300 mg day) with levofloxacin (500 mg day); this also gave good results (91%, versus 90% with the quadruple therapy). In this line, levofloxacin-based triple therapy is a novel and promising alternative that seems to offer advantages over quadruple therapy [29].

To date, only two studies have described an eradication rate comparable to ours. Quasim [30], in a series of 34 patients included prospectively after two failures, obtained eradication in only 38% of the cases, and the second one, reported by Gisbert (31), achieved -with a rifabutin-based third line therapy- only an eradication rate of 44%. These studies, together with ours, are the only ones to obtain such a disappointing outcome.

Therefore, we are faced with the first clinical trial to obtain unsatisfactory results using a rifabutin-based therapy as second line treatment. We can find no reasonable explanation for the results obtained. In the randomisation of our patients, the group treated with the quadruple therapy included more ulcerous patients than did the rifabutin group. Patients with a gastroduodenal ulcer seemed to respond better to the eradication treatment than did those with chronic gastritis [32]. This might be an explanation but our patients with a gastroduodenal ulcer had a response rate (56.8%) similar to that of the non-ulcerous ones (57.7%), and so this bias in the randomisation does not seem to have influenced the results obtained.

A priori, nor should the use of amoxicillin in this regimen have had a negative impact, as a failed eradication treatment does not increase the risk of secondary resistance to this antibiotic. Not having performed an antibiogram study before inclusion of the patients might represent a limitation of the present study, but it seems to have been proved that resistance to clarithromycin and metronidazole does not influence the effectiveness of rifabutin-based treatments [16,26,27], and primary resistance to rifabutin is minimal. Moreover, amoxicillin presents a very low rate of primary resistance in our geographical area [33].

One possible explanation for our results, compared with those of other authors, would be the duration of the treatment. Perhaps 7 days is not sufficient for a rifabutin-based therapy. Nevertheless, our study was designed for a similar duration in both groups, and guidelines recommended quadruple-based therapy for seven days [1]. Other possible explanations might be related to differences obtained in different geographical areas, as recently reported by Gisbert [31] in a study conducted in Spain.

The most plausible explanation, nevertheless, would be the amount of drug dose that was administered. As Borody reported recently [28], a course of higher doses of amoxicillin (1 gr or 1.5 gr tid) and pantoprazole (80 mg tid) for 12 days, even with low dose of rifabutin (150 mg daily), was well-tolerated and proved to be highly effective as a rescue therapy for patients having failed previous treatment. Therefore standard dose of amoxicillin (1gr bid) and PPI's (omeprazole 20 mg bid) might not be sufficient as a rescue therapy, and a better dosage should be sought in future comparative studies.

As is the case in other series that have been described, we found that quadruple therapy frequently provokes side effects that produce a reduction in the fulfilment of the therapy (11% of the patients included in the quadruple therapy regimen did not complete the recommended programme). Of the total of 14 events classified as serious or acute, most of them (11 events) corresponded to the quadruple therapy regimen. The rifabutin treatment, on the other hand, was well tolerated, and its side effects were mainly classified as mild, and had no consequences on the final rate of fulfilment of the treatment programme.

## Conclusion

In summary, in the present study a rifabutin-based therapy, associated to standard dose of amoxicillin and omeprazole applied for 7 days was not found to be effective as a second line rescue treatment against HP infection. The difficulty in administration and the frequency of side effects associated with quadruple therapy treatment underline the need to search for alternative options, with a different dose of rifabutin-based triple therapy or with alternative antibiotics which should be better tolerated, easier to administer and which match or improve upon the effectiveness achieved with this reference regimen.

## Competing interests

The author(s) declare that they have no competing interests.

## Authors' contributions

All authors contributed to the design of the study. Revision of the different versions of the study protocol: JMN, NF, FLS, EC, MC, LMR, EPM. Substantial contributions to the conception and design of the digital data record: JMN, NF, FLS, EC, MC, LMR, RR, EU, FV, IM, EPM. Acquisition of data and quality control: JMN, NF, FLS, EC, MC, LMR, RR, EU, FV, IM, EPM, JLM. Analysis and interpretation of data: JMN, EPM, FRR. All authors have read and approved the final manuscript.

## Pre-publication history

The pre-publication history for this paper can be accessed here:


